# Non-Conventional Processing Technologies in Meat and Meat Products: Toward Clean-Label, Quality, and Sustainable Innovation

**DOI:** 10.3390/foods15162874

**Published:** 2026-08-17

**Authors:** Manoela Maciel dos Santos Dias, Gabriela Aparecida Nalon, Viviane Lopes Pereira, Danielly Aparecida de Souza, Jeferson Silva Cunha, Hiasmyne Silva de Medeiros, Bruno Ricardo de Castro Leite Júnior

**Affiliations:** Department of Food Technology, Federal University of Viçosa, Av. Peter Henry Rolfs, s/n, Campus University, Viçosa 36570-900, MG, Brazil; manoela.santos@ufv.br (M.M.d.S.D.); gabriela.nalon@ufv.br (G.A.N.); viviane.pereira@ufv.br (V.L.P.); danielly.souza@ufv.br (D.A.d.S.); jeferson.cunha@ufv.br (J.S.C.); hiasmyne040485@gmail.com (H.S.d.M.)

**Keywords:** emerging food technologies, cold plasma, high hydrostatic pressure, ultrasound, microwave processing, ohmic heating, food preservation, clean label

## Abstract

The growing demand for clean-label, high-quality, and sustainable meat products has increased interest in non-conventional processing technologies as alternatives to conventional processing methods. Therefore, this review aims to critically evaluate the technological advances, practical benefits, limitations, and industrial implementation potential of cold plasma, high hydrostatic pressure, ultrasound, microwave processing, and ohmic heating in meat and meat products. Studies published between 2016 and 2026 were analyzed, with emphasis on mechanisms of action, effects on physicochemical and microbiological properties, processing performance, and evidence of industrial applicability. Current evidence indicates that these technologies have progressed beyond laboratory-scale investigations in several applications, with HHP showing the highest level of commercial adoption, particularly in ready-to-eat meat products, while ultrasound, cold plasma, microwave processing, and ohmic heating exhibit different degrees of pilot- and industrial-scale development depending on the application. These technologies can enhance microbial safety, improve techno-functional properties, optimize processing efficiency, and contribute to shelf-life extension while reducing reliance on synthetic additives. However, their effectiveness is strongly influenced by processing conditions, product composition, economic feasibility, and technology-specific limitations. Reported challenges include lipid oxidation, color deterioration, texture modifications, heating non-uniformity, high implementation costs, limited process standardization, and regulatory uncertainties. Overall, recent advances demonstrate meaningful progress toward industrial application, but the degree of technological maturity varies substantially among technologies and applications. Further research should prioritize industrial-scale validation, process standardization, techno-economic assessment, regulatory harmonization, and consumer acceptance to facilitate broader commercial adoption.

## 1. Introduction

The growing consumer demand for high-quality, minimally processed, and clean-label foods has intensified the need for innovative processing strategies capable of preserving product integrity while ensuring safety and extending shelf life [1]. In this context, emerging processing technologies, including ultrasound, high hydrostatic pressure (HHP), cold plasma, microwave processing, and ohmic heating, have gained increasing attention as viable alternatives or complements to conventional thermal methods. These technologies operate through distinct physical mechanisms, such as pressure-induced structural modifications, electromagnetic energy absorption, acoustic cavitation, and reactive species generation, enabling targeted alterations at molecular and microstructural levels. As a result, they offer significant advantages, including improved physicochemical and techno-functional properties, enhanced microbial inactivation with reduced thermal damage, and greater process efficiency [2,3].

It is noteworthy that the large-scale implementation of emerging technologies in the meat industry still faces significant challenges, including high capital investment, lack of standardized operational frameworks, technological limitations related to process uniformity and efficiency, and issues associated with consumer acceptance. Additionally, regulatory uncertainties and the need for robust safety and efficacy validation further constrain industrial adoption. Therefore, continued technological optimization, standardization, and interdisciplinary collaboration are essential in facilitating the transition from experimental applications to reliable and economically viable industrial systems [4].

This review aims to provide an integrated and critical assessment of non-conventional processing technologies applied to meat and meat products, with particular emphasis on their technological advances, practical benefits, mechanisms of action, limitations, and potential for industrial implementation. The review examines their effects on physicochemical, techno-functional, and microbiological properties, while also considering processing efficiency, clean-label applications, technological maturity, and the technical, economic, regulatory, and consumer-related factors that influence their adoption in the meat industry.

## 2. Materials and Methods

This study was conducted as a narrative literature review addressing non-conventional processing technologies applied to meat and meat products, including cold plasma, high hydrostatic pressure, ultrasound, microwave processing, and ohmic heating with emphasis on their mechanisms of action, physicochemical and techno-functional effects, microbiological implications, and industrial applicability. The literature search was performed using the Scopus, Web of Science, ScienceDirect, PubMed, and Google Scholar databases, with emphasis on studies published between 2016 and 2026. The search strategy combined keywords and Boolean operators including “emerging processing technologies”, “non-conventional technologies in meat processing”, “meat processing”, “non-thermal technologies”, “ultrasound in meat products”, “cold plasma”, “high hydrostatic pressure in food processing”, “microwave in food processing”, “microbial inactivation”, “techno-functional properties”, “protein functionality”, “food preservation”, and “clean-label meat products”.

Original research articles were used as the primary basis for evaluating processing effects and technological performance, while review papers were also considered to provide broader technological and contextual perspectives. Studies addressing structural, physicochemical, microbiological, sensory, technological, and industrial aspects of non-conventional processing technologies were prioritized. Conference abstracts, duplicated studies, publications unrelated to meat systems, and articles lacking sufficient methodological or scientific relevance were excluded. The selected studies were critically analyzed by comparing their mechanisms of action, technological performance, effects on meat quality and safety, methodological limitations, consistency and divergence of findings, technological maturity, industrial applicability, and current research gaps. Particular attention was given to processing conditions, scale of application, and evidence supporting process scalability and industrial implementation. The findings were then organized according to the technological approach and the main outcomes reported for meat quality, process efficiency, microbial safety, sustainability, and industrial feasibility.

## 3. Cold Plasma

### 3.1. Operating Principle of Cold Plasma

**Figure 1:**
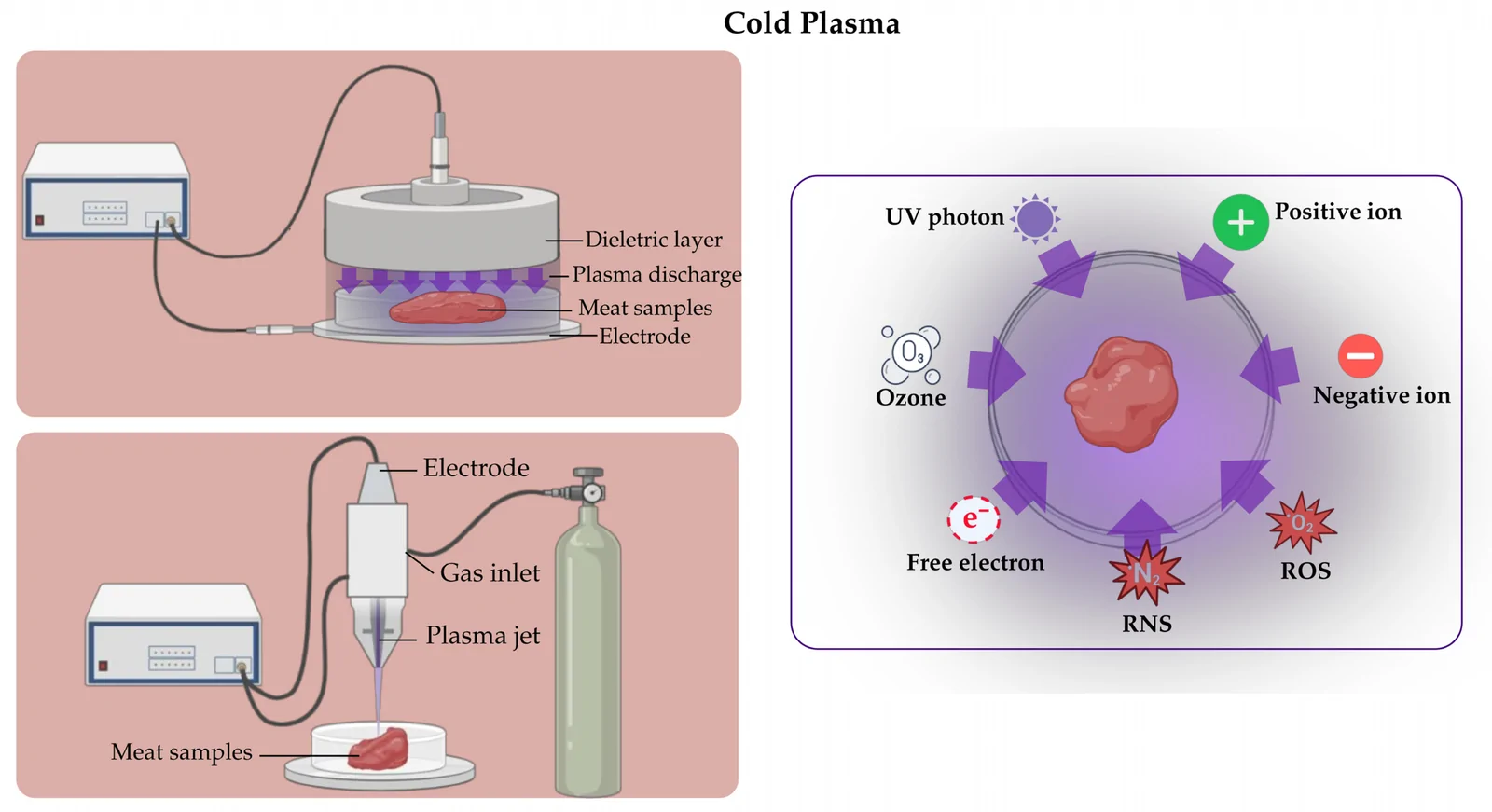
Schematic representation of the operating principle of cold plasma in meat. An electrical discharge ionizes the working gas, generating reactive oxygen and nitrogen species (ROS/RNS), ions, electrons, and UV photons, which interact with the meat surface, contributing to microbial inactivation and physicochemical changes. Purple arrows indicate the interaction of plasma-generated reactive species with the meat surface.

Cold plasma, the fourth state of matter, is generated when an electrical potential difference is applied to a gas [5,6], causing electron acceleration, ionization and a cascade that turns the gas into a conductive medium. During electron collisions, the transferred energy promotes the dissociation and excitation of gas molecules, resulting in the formation of reactive oxygen species (ROS) and reactive nitrogen species (RNS), as well as ultraviolet radiation photons, excited electrons, and ions [7,8]. This process is illustrated in Figure 1.

The choice of working gas directly influences the composition and concentration of reactive species formed during the plasma discharge, thereby impacting process efficiency and its effects on food matrices [6,9]. Among the most commonly used gases are oxygen, nitrogen, atmospheric air, and noble gases such as helium and argon. In addition, process efficiency depends on operational parameters such as applied voltage, discharge frequency, exposure time, and the distance between the electrode and the sample, as well as environmental conditions like relative humidity, all of which influence the generation and stability of reactive species. Cold plasma can be applied either directly, when the food is exposed to the reactive species generated in the plasma, or indirectly, through the use of previously treated fluids, such as plasma-activated water (PAW), thereby expanding the possibilities for technological applications [10]. Cold plasma can be applied in different stages of the meat production chain, including the treatment of carcasses, fresh cuts, ground meat, and processed products [11].

### 3.2. Effect of Cold Plasma on the Quality of Meat and Meat Products

#### 3.2.1. Effect of Cold Plasma on Sensory Properties, Water-Holding Capacity, pH, Lipid Oxidation, and Protein Structure

The application of cold plasma induces a range of modifications in the meat matrix, resulting from the interaction of reactive oxygen and nitrogen species (ROS/RNS) with its main constituents, leading to direct changes in pH, color, texture, water-holding capacity and overall quality. In general, these effects depend on processing conditions and food composition, resulting in both improvements and undesirable alterations (Table 1).

The application of cold plasma to meat products promotes structural modifications mediated by ROS/RNS, including a slight reduction in pH due to the formation of acidogenic compounds. This decline can favor tenderness but can also compromise water retention capacity when excessive. Changes in color are frequently associated with myoglobin oxidation, resulting in reduced redness in red meats and increased lightness and yellowness in white meats [17]. Furthermore, reactive oxygen and nitrogen species generated during cold plasma treatment may promote lipid and protein oxidation, as reflected by increases in TBARS and protein carbonyls and a reduction in sulfhydryl groups (Table 1). Despite this, protein modifications caused by the treatment can improve techno-functional properties, such as emulsification, highlighting the importance of adjusting operational settings to balance functionality and sensory quality.

Changes in pH are generally limited due to the buffering capacity of myofibrillar proteins. However, slight reductions may occur as a result of acidogenic compound formation and microbial degradation during storage [6]. This pH decline has been associated with the production of organic acids and may influence myofibrillar structure by increasing electrostatic repulsion between proteins, promoting filament separation and contributing to texture modifications, including meat tenderization [17]. Concurrently, as a result of the separation of filaments within the myofibrillar structure, water-holding capacity (WHC) may be enhanced, leading to greater moisture retention, directly reflecting in juiciness and higher sensory acceptance, as demonstrated in studies with chicken fillets [18,19].

Cold plasma alters meat color mainly through the oxidation of muscle pigments due to the interaction of reactive species with the system, promoting a reduction in the intensity of red coloration. Color is commonly evaluated using the CIELAB color space, in which L*, a*, and b* represent lightness, the red–green coordinate, and the yellow–blue coordinate, respectively [19]. The decrease in the a* value is directly associated with the oxidation of oxymyoglobin into metmyoglobin. In addition, increasing the processing voltage of this non-conventional technology can intensify this loss of red color [18,19]. Furthermore, changes in lightness (L*) and yellowness (b*) parameters may also occur. The b* value tends to increase due to the accumulation of metmyoglobin on the meat surface, while the L* value tends to remain stable. However, in white meats, such as chicken, an increase in L* and b* parameters may occur, especially when oxygen is used as the working gas [18,19].

In the lipid context, cold plasma treatment alters meat characteristics mainly by inducing oxidation and modifying the structure of fatty acids, mediated by reactive oxygen and nitrogen species formed during the process. Cold plasma tends to accelerate the production of lipid peroxides, resulting in an increase in thiobarbituric acid reactive substances (TBARS) and malondialdehyde (MDA) values, as observed in beef burgers treated at 70 kV, where oxidation increased from 0.04 to 0.16 mg MDA/kg immediately after treatment [17]. Recent studies have shown that oxidation of unsaturated fatty acids is frequently observed, resulting in the formation of secondary compounds that may compromise oxidative stability, leading to rancidity and consequently impacting consumer sensory perception [8]. However, proper control of parameters can minimize these effects, and the use of inert gases, such as argon, may help mitigate the development of oxidation [8].

At the same time, myofibrillar proteins may also undergo structural modifications, affecting techno-functional properties such as water-holding capacity and emulsification, directly influencing texture, tenderness, and juiciness of the products. Cold plasma treatment promotes molecular unfolding of myofibrillar proteins, altering their secondary and tertiary structures, exposing hydrophobic amino acid residues, and facilitating protein adsorption at the oil–water interface, thereby improving both the emulsifying activity index (EAI) and emulsion stability index (ESI), which are important indicators of techno-functional performance and contribute to the texture of processed meat products [8].

In addition to physicochemical changes, sensory evaluation has been widely used to understand the impacts of cold plasma on the overall quality of meat and meat products. In studies using dielectric barrier discharge (DBD), it has been observed that short treatments (3–5 min) can promote significant improvements in attributes such as flavor, texture, and overall acceptance, in both raw and cooked samples. However, reductions in appearance and color attributes have been reported in treatments performed with oxygen, while samples treated with inert gases, such as argon, tend to show better sensory performance, possibly due to the lower induction of oxidative processes [12].

#### 3.2.2. Effect of Cold Plasma on Microbial Decontamination of Meat Products

The efficiency of cold plasma as a non-conventional technology for improving the microbiological quality of meat and meat products has attracted increasing interest in recent years. This technology can be applied through different configurations and operational strategies, allowing its adaptation to the specific characteristics of the meat matrix and the processing objectives. The effect of cold plasma on the microbiological quality of meat products is presented in Table 2.

Cold plasma has demonstrated the ability to reduce populations of pathogens and spoilage microorganisms, with the extent of microbial inactivation depending mainly on exposure time and treatment intensity (Table 2). Gram-negative bacteria tend to show greater susceptibility due to the lower structural protection of their cell walls. Furthermore, the technology exhibits a synergistic effect when combined with other obstacles, such as organic acids, essential oils, and ultrasound, enhancing the antimicrobial action. These results also indicate an evolution from direct applications to indirect approaches, such as plasma-activated water and brines, which favor industrial integration and maintain microbiological efficacy with less oxidative impact on the product.

Microbial inactivation is strongly influenced by treatment time and applied voltage. In chicken breast, increasing the treatment time from 1 to 5 min resulted in significantly greater reductions in spoilage microorganisms [13]. Gas composition also plays a crucial role. Studies using DBD have shown that the presence of oxygen enhances inactivation due to the greater generation of ozone and reactive species [14]. However, the use of noble gases, such as argon, has been shown to be more efficient than pure oxygen at low voltages (20 kV), achieving reductions greater than 2 log CFU/g in chicken [13,18].

**Figure 2:**
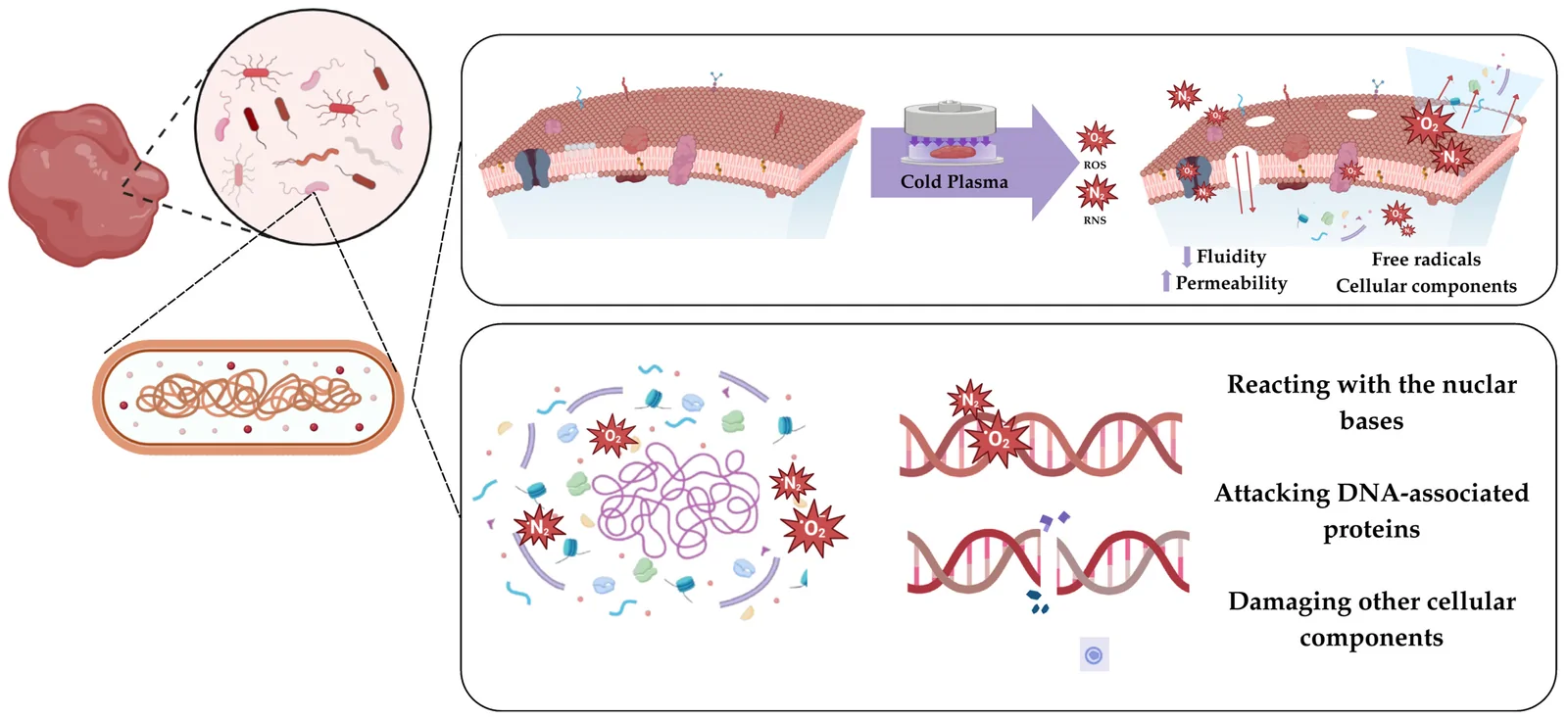
Effects of cold plasma on microorganisms present in meat. Cold plasma generates reactive oxygen species (ROS) and reactive nitrogen species (RNS), which interact with microbial cell membranes and intracellular components, increasing membrane fluidity and permeability and causing damage to cellular components and DNA. Purple arrows indicate the effects induced by plasma-generated reactive species, whereas ROS and RNS are represented by red symbols.

Regarding microbial structures, there is a tendency for greater susceptibility in Gram-negative bacteria compared to Gram-positive ones when exposed to cold plasma. This is attributed to differences in the thickness of the peptidoglycan layer, which provides greater mechanical resistance and protection against reactive species [22]. In the same context, the loss of cell viability occurs mainly through the generation of hydroxyl radicals, hydrogen peroxide, nitric acid, and ozone, which attack cellular macromolecules. Additionally, the modification of structural proteins and the inactivation of essential enzymes result in microbial inactivation, as reactive species can cause irreversible genetic damage [12]. The effects of cold plasma on microorganisms present in meat are shown in Figure 2.

The effectiveness of cold plasma is maximized when combined with other technologies, creating a synergistic effect. The combination of plasma-activated water with ultrasound accelerates the thawing process and enhances microbial reduction, making the meat surface more porous and facilitating the action of reactive species [12]. In addition, the use of plasma-activated acetic acid (PAAA) has demonstrated a superior bactericidal effect against *Salmonella* compared to the use of acid alone, taking advantage of pH reduction to destabilize cell membranes [16].

Recent advances in the application of cold plasma in the meat industry indicate that its potential extends beyond decontamination, including improvements in the techno-functional properties of meat products [6]. Practical benefits include meat tenderization through alterations in myofibrillar structure and pH, as well as improvements in emulsion stability and emulsifying activity resulting from protein modification [17]. Industrial integration may be facilitated through plasma-activated fluids, such as plasma-activated water, which can be incorporated into existing operations including washing, curing, and thawing [10,15]. Furthermore, recent studies have explored cold plasma in hurdle systems, including combinations with ultrasound, to enhance microbiological safety while maintaining sensory quality [12]. These developments indicate that cold plasma is progressing beyond proof-of-concept applications, although further pilot- and industrial-scale validation is required to establish its economic and operational feasibility.

## 4. High Hydrostatic Pressure (HHP)

### 4.1. Effect of High Hydrostatic Pressure on the Quality of Meat and Meat Products

Among the non-conventional processing technologies discussed in this review, HHP is one of the most commercially established, with particularly strong adoption in ready-to-eat (RTE) meat products, where it is mainly used for post-packaging microbial control and shelf-life extension [23]. High hydrostatic pressure (HHP) technology, also known as high isostatic pressure (HIP) or high-pressure processing (HPP), selectively acts on non-covalent interactions, enabling the modulation of the structural and functional properties of meat [24]. At the molecular level, pressure mainly affects non-covalent interactions, including hydrogen bonds, hydrophobic interactions, and ionic bonds, while covalent structures remain largely preserved [24,25]. Therefore, HHP promotes conformational changes in muscle proteins, influencing the technological functionality and microbiological safety of meat products. Myofibrillar proteins, particularly myosin and actin, are among the most pressure-sensitive components in muscle systems. At moderate pressures (~150–250 MPa), partial unfolding occurs due to destabilization of hydrophobic regions, exposing reactive domains and increasing protein surface reactivity [26,27]. At higher pressures (> 400 MPa), aggregation becomes more pronounced as unfolded proteins interact via hydrophobic associations, accompanied by reduced solubility [28]. These structural transitions modify muscle tissue organization and directly affect the functional properties of meat systems.

Evidence indicates that HHP-induced changes occur through progressive structural transitions rather than fixed thresholds, ranging from predominantly reversible unfolding below 300 MPa to aggregation-dominated and largely irreversible modifications above 500 MPa [24,25]. These pressure-dependent effects are closely linked to water redistribution within muscle systems. Partial unfolding can enhance water-binding capacity through exposure of hydrophilic groups, whereas extensive aggregation may restrict water mobility or promote exudation, depending on network density [29].

**Figure 3:**
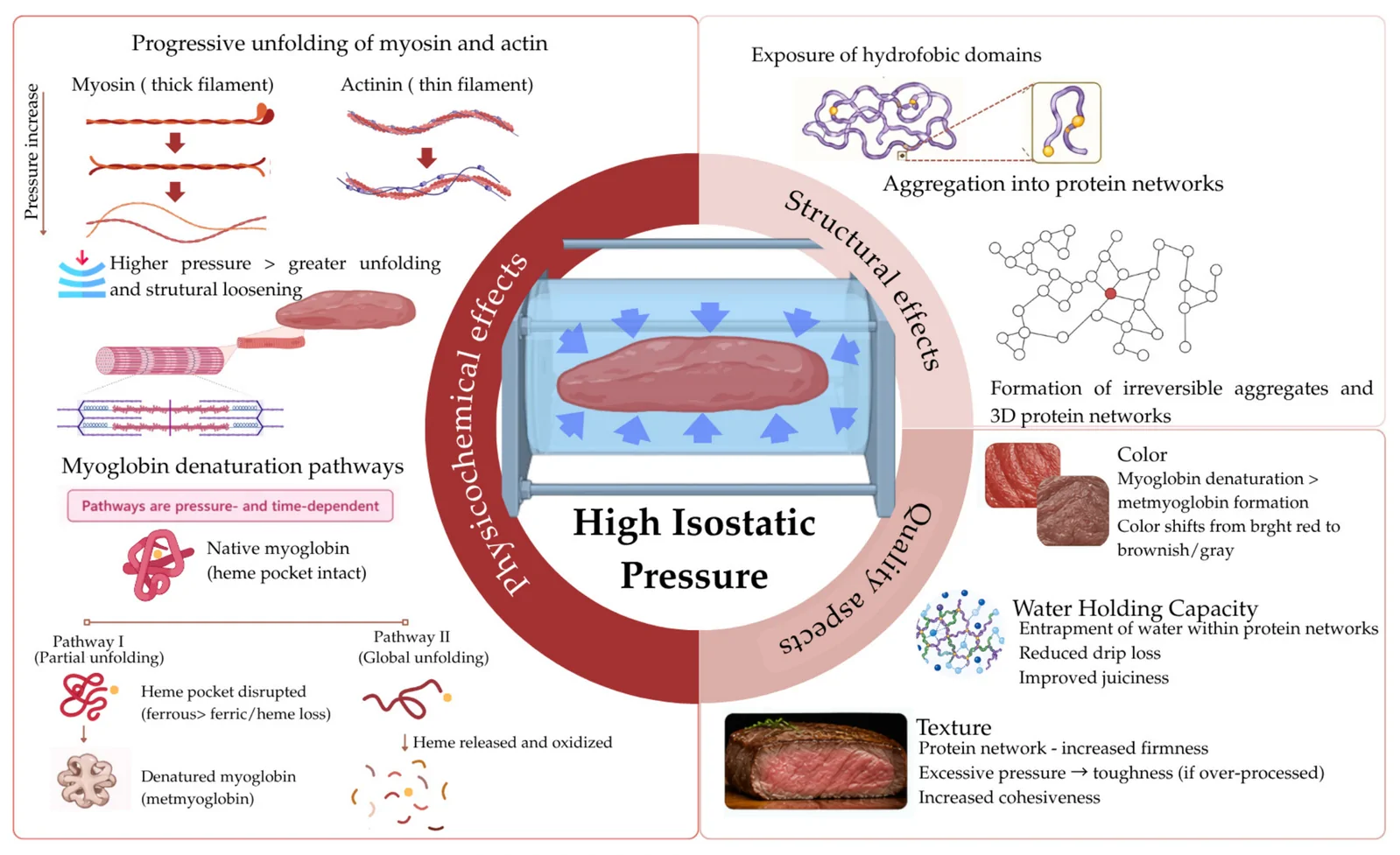
Mechanistic effects of HHP on muscle systems, integrating pressure-induced protein transitions, water redistribution, and their implications for structural and functional properties.

The mechanistic effects of HHP on muscle systems, integrating pressure-induced protein transitions, water redistribution, and their implications for structural and functional properties are summarized in Figure 3.

Overall, HHP effects on meat systems arise from the interplay between protein conformational changes and water redistribution. As pressure increases, the transition from partial unfolding to aggregation alters protein organization, influencing texture, water-holding capacity, and structural integrity [24,25], which will be further discussed below.

### 4.2. Effect of High Hydrostatic Pressure on Water-Holding Capacity and Sensory Quality

HHP-induced changes in meat systems are governed by pressure-driven structure–function relationships, in which conformational transitions of muscle proteins influence WHC, texture, color stability, and lipid oxidation. As summarized in Table 3, these effects vary according to meat matrix, pressure intensity, and the balance between protein unfolding, aggregation, and structural disruption.

As summarized in Table 3, protein denaturation generally progresses from partial unfolding at moderate pressures to aggregation at higher pressures. In pork systems treated at 200 MPa, increased hardness has been associated with the formation of denser protein networks [27], whereas in wild red deer meat treated at 500 MPa, pressure-induced structural disruption was associated with increased tenderness [28]. These contrasting responses indicate that texture modifications under HHP are matrix-dependent and cannot be explained solely by the applied pressure. Similarly, WHC is also highly sensitive to pressure-induced protein rearrangements. Moderate pressures may enhance water retention through exposure of hydrophilic groups, while higher pressures can either improve or impair water retention depending on the organization of the resulting protein network [26,31].

Color changes remain one of the major limitations of HHP application in meat products. Pressure-induced modifications in myoglobin structure contribute to increased lightness and reduced redness, potentially affecting consumer acceptance in products where fresh appearance is critical [33]. In addition, lipid oxidation may also be intensified during storage due to pressure-induced structural modifications that increase lipid exposure to oxidative reactions. In sliced Iberian dry-cured *salchichón*, HHP reduced microbial counts but increased oxidative changes during storage [32].

Unlike thermal processing, HHP better preserves the sensory characteristics of meat because it primarily acts on non-covalent interactions of biomolecules without relying on high temperatures. This minimizes the changes typically associated with thermal processing, supporting its application in clean-label meat products [30,33].

Overall, the physicochemical and techno-functional effects of HHP are highly dependent on the interaction between pressure conditions and meat matrix characteristics. Therefore, HHP should be considered not only as a preservation technology but also as a processing tool capable of tailoring meat functionality according to the desired product attributes.

### 4.3. Effects of High Hydrostatic Pressure on Microbiological Quality

HHP is an effective non-thermal strategy for microbial inactivation in meat systems, although its efficacy depends on pressure intensity, treatment time, and food matrix characteristics. Moderate pressures (~200 MPa) are generally associated with partial reductions in spoilage microbiota [27], whereas higher pressures (≥600 MPa) can achieve substantial inactivation of pathogens such as *Listeria monocytogenes* and *Salmonella* under appropriate processing conditions [34]. Increasing pressure intensity promotes a progressive transition from sublethal injury to irreversible cellular damage, with membrane disruption and metabolic collapse becoming predominant at pressures exceeding 400 MPa [25,35].

Microbial inactivation under HHP is mainly associated with disruption of cell membrane integrity, protein denaturation, and metabolic impairment, which collectively compromise microbial viability [36]. However, microbial resistance varies according to cellular structure, physiological state, and the protective effects of the food matrix [36].

One of the main limitations of HHP is its reduced effectiveness against bacterial spores, which are considerably more pressure-resistant than vegetative cells. Consequently, HHP alone may be insufficient in products where spore-forming microorganisms are relevant. Therefore, the combination of HHP with other preservation approaches has demonstrated potential to enhance microbial inactivation. In particular, natural antimicrobials such as nisin and plant-derived compounds may act synergistically with pressure treatment, possibly due to increased membrane permeability induced by HHP [37]. Overall, the microbiological effectiveness of HHP depends on the interaction between processing conditions, microbial characteristics, and matrix effects. Thus, processing parameters must be optimized to ensure microbial safety while maintaining product quality.

From an industrial perspective, HHP can be adjusted according to the intended application. Moderate pressures are commonly associated with shelf-life extension through reduction in spoilage microbiota, whereas higher pressures are required when microbial safety is the primary objective [25,26]. This flexibility allows HHP to be adapted to different meat products and quality requirements. HHP also demonstrates compatibility with hurdle technologies. The combination of pressure treatment with mild thermal processes or natural antimicrobials has shown potential to enhance microbial control while maintaining product quality, supporting clean-label preservation strategies [37,38].

The studies reviewed in Section 4.1, Section 4.2 and Section 4.3 encompass diverse matrices, including fresh whole muscle [26,28,29], comminuted products [30,33], dry-cured fermented products [32], and plant-based formulations [37]. Pressure intensity and matrix composition jointly influence the functional response to HHP: moderate pressures (~200 MPa) increased firmness in comminuted pork systems [26,29], whereas higher pressures (~500 MPa) increased tenderness in fresh deer muscle [28], a divergence attributable to differences in myofibrillar composition and connective tissue content. Color alteration, characterized by increased lightness and reduced redness, was among the most consistently reported responses across matrices [32,33,37], although Timón et al. [33] reported no perceivable impact in cooked products. Most studies evaluated a limited range of pressure–time combinations within individual matrices, restricting the establishment of transferable dose–response relationships. HHP combined with natural antimicrobials has shown synergistic potential for the inactivation of *Listeria monocytogenes* [34,37], although systematic validation across different pathogen groups, food matrices, and processing conditions remains limited. Bacterial spore resistance and the need for validated process conditions and regulatory criteria remain important challenges for broader standardization [36]. Future research should prioritize cross-matrix experimental designs, systematic evaluation of hurdle strategies, and integration of validated consumer acceptance data.

### 4.4. Industrial Applicability of HHP

HHP has achieved a relatively high level of industrial maturity among non-conventional processing technologies, with commercial systems operating in several regions worldwide and meat products representing an important application area [36]. Its commercial use is particularly established for post-packaging treatment of RTE meat products, where HHP is primarily applied to enhance microbiological safety and extend shelf life while maintaining product quality [36,37].

Documented applications include delayed growth of *Listeria monocytogenes* following post-packaging treatment combined with natural antimicrobials (600 MPa, 3 min) [37], as well as reductions in phosphate and sodium contents while maintaining desirable product characteristics [31,33]. These applications illustrate the potential of HHP to support clean-label reformulation strategies in addition to its established role in microbial control.

Despite its relatively advanced industrial status, HHP remains associated with important economic and operational challenges. High capital investment and the batch nature of conventional HHP equipment can increase processing costs compared with established thermal technologies [36]. In addition, pressure-induced changes in color and texture may limit its application to some products, particularly when high pressures are required for microbial safety [36,37]. Consumer acceptance and product-specific optimization therefore remain important considerations for expanding the use of HHP across different meat categories.

Overall, HHP currently represents one of the most industrially mature non-conventional processing technologies considered in this review. Nevertheless, broader adoption will benefit from continued process standardization, optimization of pressure–time conditions, techno-economic assessment, regulatory alignment, and further validation of consumer acceptance [36,37].

## 5. Ultrasound

### 5.1. Operating Principle of Ultrasound

Ultrasound technology is based on the propagation of mechanical waves at frequencies above 20 kHz through a medium. In food processing, high-intensity ultrasound (20–100 kHz; >1 W/cm^2^) is the most commonly applied modality due to its ability to generate acoustic cavitation, the fundamental phenomenon responsible for ultrasound-induced modifications in food systems [2,39].

Acoustic cavitation involves the formation, growth, and implosive collapse of microscopic bubbles generated by alternating compression and rarefaction cycles of the acoustic wave. Bubble collapse produces localized extreme conditions, including high temperatures, pressures, shock waves, microjets, and microstreaming, which generate both mechanical and sonochemical effects. These phenomena promote structural disruption, enhance mass transfer, and generate reactive species capable of interacting with proteins, lipids, and microbial cells. Consequently, cavitation represents the physicochemical basis underlying the technological, structural, and microbiological effects of ultrasound in meat and meat products [40,41].

**Figure 4:**
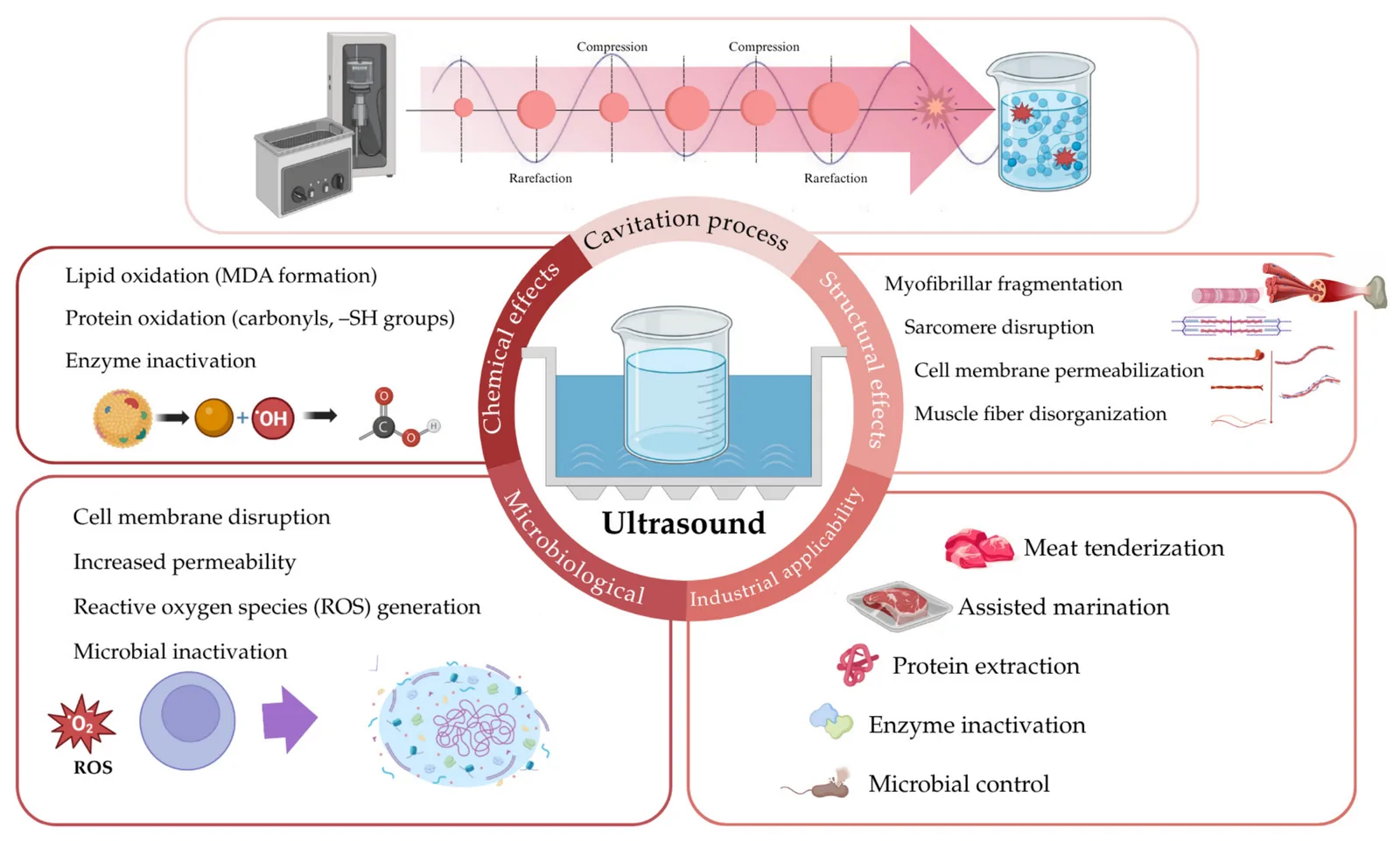
Schematic representation of ultrasound application in meat systems, highlighting the cavitation process generated by acoustic waves and its associated effects.

The acoustic cavitation phenomenon and the resulting mechanical and sonochemical events responsible for ultrasound-induced modifications in meat systems are illustrated in Figure 4.

These cavitation-mediated interactions form the physicochemical basis of the structural and functional changes discussed in the following sections.

### 5.2. Effects of Ultrasound on Techno-Functional Properties and Flavor of Meat Products

The cavitation phenomena described above directly influence the structural organization and functionality of muscle proteins, resulting in important modifications in the techno-functional properties of meat systems. Myofibrillar proteins, particularly myosin and actin, are among the main targets of ultrasound-induced structural changes. Cavitation-generated mechanical forces promote partial protein unfolding, exposing previously buried hydrophobic and sulfhydryl groups while reducing aggregate size and increasing protein dispersion. These molecular rearrangements generally enhance protein solubility and reactivity, thereby improving their functional performance in meat systems [42].

The modification of protein structure has direct implications for water retention and texture. The exposure of hydrophilic groups increases protein–water interactions, improving water-holding capacity, and reducing cooking and drip losses. Consequently, ultrasound-treated meat products often exhibit improved yield, juiciness, and technological performance [42,43]. In addition, cavitation-induced disruption of muscle fibers, connective tissue components, and myofibrillar organization reduces structural resistance, contributing to lower shear force values and enhanced tenderness [44].

Ultrasound may also improve the emulsifying properties of meat proteins by increasing surface hydrophobicity, exposing functional groups, and reducing particle size. These modifications facilitate protein adsorption at oil–water interfaces and promote the formation of smaller and more stable droplets, enhancing emulsion stability and the quality of processed meat products. However, excessive ultrasound intensity or prolonged treatment may induce protein aggregation and accelerate oxidative reactions, potentially compromising functionality, color stability, and sensory quality [45].

Beyond its effects on texture and protein functionality, ultrasound can influence flavor development by accelerating biochemical reactions associated with the generation of aroma precursors. Cavitation may enhance the release and activity of endogenous enzymes, including proteases and lipases, promoting the formation of peptides, free amino acids, and fatty acids that participate in flavor generation. Furthermore, ultrasound can intensify lipid oxidation and Maillard-related reactions, contributing to the formation of volatile compounds such as aldehydes, ketones, sulfur-containing compounds, and heterocyclic molecules that influence meat aroma, particularly after thermal processing. While moderate ultrasound treatments may enhance the formation of desirable aroma precursors and flavor-active compounds, excessive cavitation can accelerate lipid oxidation and increase the formation of off-flavor compounds, highlighting the need for careful optimization of processing conditions [46,47].

The structural and functional modifications induced by acoustic cavitation have expanded the application of ultrasound across diverse meat-processing operations. The principal applications, their underlying mechanisms, technological effects, and potential industrial benefits are summarized in Table 4.

As shown in Table 4, ultrasound applications extend throughout the meat production chain, from raw material processing to product formulation and sanitation. Most reported benefits originate from cavitation-mediated enhancement of heat and mass transfer phenomena, which improve process efficiency and product quality while reducing processing times. Beyond traditional applications such as tenderization and marination, recent studies have expanded the use of ultrasound toward protein recovery, valorization of meat by-products, and development of low-fat formulations. Collectively, these applications demonstrate the versatility of ultrasound across different processing operations, reinforcing its potential to improve process efficiency, product quality, and resource utilization in meat systems.

Overall, the effects of ultrasound are strongly dependent on processing parameters, including frequency, intensity, treatment time, temperature, and matrix composition. Therefore, optimization of operational conditions is essential to maximize technological benefits while minimizing undesirable structural and oxidative changes.

### 5.3. Effects of Ultrasound on Microbial Control

Among the various industrial applications of ultrasound, microbial control has received particular attention due to its potential to improve the microbiological safety and shelf life of meat products. Acoustic cavitation generates physical, mechanical, and chemical effects capable of inactivating spoilage and pathogenic microorganisms while minimizing quality deterioration associated with conventional thermal treatments [2,46].

The antimicrobial activity of ultrasound is primarily associated with cavitation-induced structural damage to microbial cells. Membrane disruption, increased permeability, protein denaturation, leakage of intracellular constituents, and eventual cell lysis are frequently reported. Simultaneously, cavitation promotes the formation of reactive species, such as hydroxyl radicals (OH), which oxidatively damage lipids, proteins, and nucleic acids, leading to progressive loss of cellular integrity and functionality [60].

Ultrasound has demonstrated efficacy against several microorganisms relevant to meat systems, including *Escherichia coli*, *Salmonella* spp., *Listeria monocytogenes*, and *Staphylococcus aureus*. However, microbial inactivation is strongly influenced by microbial physiology and structural characteristics. Gram-negative bacteria are generally more susceptible due to their thinner cell envelope, whereas Gram-positive bacteria exhibit greater resistance because of their thick peptidoglycan layer. Likewise, bacterial spores represent the most resistant forms owing to their highly protective structure [61].

Despite its antimicrobial potential, ultrasound applied as a standalone treatment generally results in only moderate microbial reductions in complex meat matrices. Consequently, combined preservation approaches have received increasing attention. Thermosonication, ultrasound-assisted cold plasma, and combinations with natural antimicrobials have demonstrated synergistic effects by enhancing membrane permeability, facilitating oxidative damage, disrupting biofilms, and improving overall microbial inactivation efficiency while reducing the intensity of thermal treatments [3,62].

In addition to its direct effects on microorganisms present in meat products, ultrasound has also demonstrated considerable potential as a cleaning and sanitization technology in meat processing facilities. Acoustic cavitation generates microjets, localized shear forces, and turbulent flow conditions capable of disrupting and removing biofilms, cellular debris, and organic residues adhered to food-contact surfaces. These mechanisms improve cleaning efficiency, enhance industrial hygiene, and may contribute indirectly to reducing cross-contamination risks within processing environments. Consequently, ultrasound-assisted cleaning has emerged as a promising sustainable alternative or complementary strategy to conventional sanitation procedures in the meat industry [63].

### 5.4. Industrial Implementation of Ultrasound

Ultrasound has progressed beyond laboratory-scale research for several food-processing applications, with commercial and pilot-scale systems available for operations such as cleaning, extraction, emulsification, marination, tenderization, thawing, drying, and process intensification [49,64]. In meat processing, however, the degree of technological maturity varies considerably according to the application. The greatest industrial potential appears to be associated with process intensification, particularly marination, thawing, tenderization, drying, and mass-transfer enhancement, whereas the use of ultrasound as a stand-alone preservation technology remains less mature.

Recent research has therefore shifted from demonstrating the effects of acoustic cavitation toward process optimization, reactor design, continuous-flow systems, energy efficiency, and scale-up. Nevertheless, heterogeneous meat matrices pose challenges for achieving homogeneous acoustic energy distribution, while the lack of standardized operating protocols, limited pilot-scale validation, uncertain techno-economic performance, and the scarcity of continuous-flow systems remain barriers to broader industrial adoption. Future studies should prioritize pilot- and industrial-scale validation, reactor engineering, process monitoring, techno-economic assessment, and integration with complementary preservation technologies.

## 6. Microwave

### 6.1. Operating Principle of Microwave Heating

Microwave radiation consists of electromagnetic waves with frequencies typically of 915 MHz and 2.45 GHz, enabling rapid and efficient volumetric heating of foods [65]. This technology can modify the structure of macromolecules and induce physicochemical changes, which has led to its widespread use in both industrial applications and scientific research [66].

**Figure 5:**
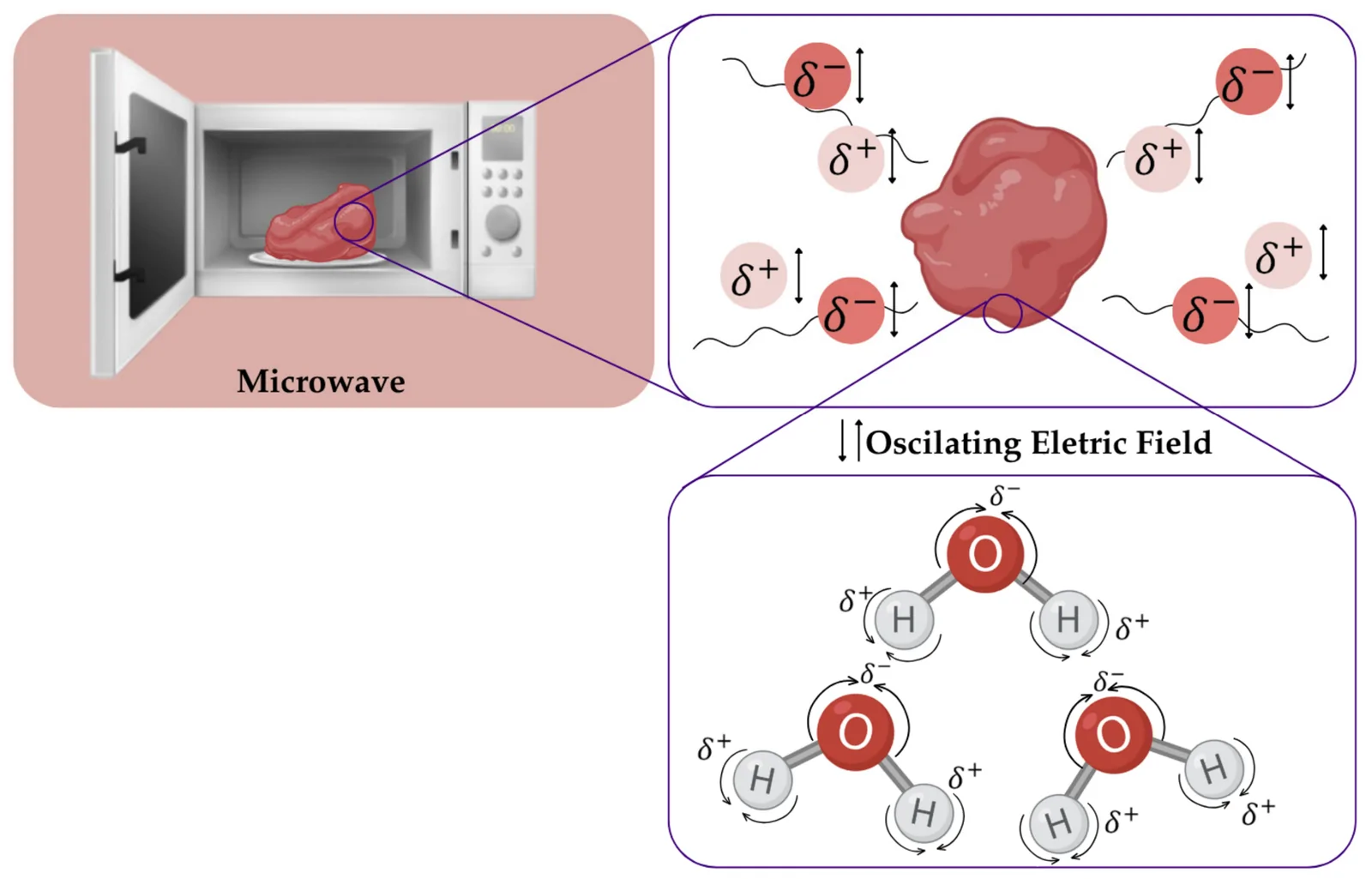
Schematic illustration of the mechanisms involved in the microwave heating of meat samples. The arrows indicate the oscillating electric field and the associated movement and reorientation of polar molecules within the meat matrix.

Microwaves’ heating occurs through the absorption of electromagnetic energy and its conversion into heat, primarily through dipolar rotation and ionic conduction [67] (Figure 5). In dipolar rotation polar molecules, such as water, align with the alternating electric field, resulting in rapid oscillations that generate heat through molecular friction [68]. In contrast, ionic conduction involves the movement and collisions of ions (such as Na^+^ and Cl^−^) present in the food matrix, which dissipate energy as heat [67].

Microwave heating efficiency depends on the dielectric properties of food, which are influenced by food composition, temperature and frequency [67]. Unlike conventional heating, microwave heating is volumetric, with energy absorbed directly throughout the food matrix, enabling faster processing but also favoring localized overheating (hot spots) [69]. Additionally, penetration depth affects heating uniformity and overall process efficiency.

### 6.2. Effects of Microwave Heating on Physicochemical, Sensory, Techno-Functional Properties, and Cooking Time

The interaction of microwave energy with polar molecules can markedly influence the physicochemical and techno-functional properties of meat products. These effects depend on multiple factors, including processing conditions and product characteristics. In this context, studies on the application of microwave technology in meat systems, emphasizing processing conditions and the main effects reported in the literature are compiled in Table 5.

The results presented in Table 5 indicate that microwave exposure times exceeding 2 min were consistently associated with quality deterioration, including reduced water-holding capacity, increased hardness, and enhanced lipid oxidation. In contrast, shorter treatments (<90 s) promoted desirable effects, such as increased nucleotide and free amino acid contents, suggesting improved flavor development. The substantial weight losses reported for yak meat (37–45%) and the progressive mass losses observed in beef with increasing cooking time further indicate that moisture migration becomes a dominant phenomenon during prolonged microwave heating.

Among these changes, the reduction in water-holding capacity appears to be one of the most consistently reported consequences of microwave processing and is closely associated with the overall quality of meat products. A recent study conducted by Kaplan et al. [75] observed that microwave processing significantly reduced cooking time of beef; however, it resulted in greater mass loss (42.4%) compared to conventional heating, indicating a reduction in water-holding capacity. This behavior is directly related to the rapid denaturation of myofibrillar proteins, which leads to structural contraction and the expulsion of water from the meat matrix. Similar results were reported by Gao et al. [76], where microwave treatment reduced cooking time of pork and beef (longissimus dorsi muscles) by 4.7 times compared to conventional boiling. Furthermore, microwave technology was associated with excessive protein denaturation, resulting in increased shear force (tougher meat), as well as reduced moisture content and changes in chemical composition. Changes in color parameters were also observed, with increases in L* and b* parameters and decreases in a*. Indeed, microwave heating can accelerate protein unfolding, promoting a series of structural changes, including the rupture of disulfide bonds, increased exposure of sulfhydryl groups, modifications in the proportion of ordered and disordered structures, and increased hydrophobicity [77].

Although comparative studies indicate that wet-heating methods, such as steam cooking, generally provide superior texture preservation and water retention compared to microwave heating, Xu et al. [74] demonstrated that microwave-assisted salting improved the water-holding capacity and partially enhanced the texture of beef. These effects were attributed to enhanced salt diffusion and controlled protein modifications. Furthermore, microwave treatment promoted the formation of unique aromatic compounds, highlighting its potential to improve sensory characteristics. In addition, the response of meat to microwave heating strongly depends on the product structure. In beef, Kaplan et al. [75] observed that steaks showed greater mass loss and increased firmness and toughness under microwave treatment, while ground beef exhibited a different textural response.

Regarding oxidative stability, microwave treatments can lead to a consistent increase in lipid oxidation in meat (Table 5), resulting in the formation of hydroperoxides and secondary compounds such as aldehydes, ketones, and other volatiles responsible for rancidity. This process contributes to meat quality deterioration, affecting sensory attributes, oxidative stability, and nutritional value [78]. A recent study showed that meats subjected to microwave processing exhibited significantly higher levels of thiobarbituric acid reactive substances (TBARS) and cholesterol oxidation products [79]. Consistent with these findings, Kaplan et al. [75] reported that microwave treatment increased lipid oxidation (TBARS).

The effects of microwave heating on lipid oxidation are related to the structural changes induced in the meat matrix. Rapid heating leads to the disruption of cellular membranes, facilitating lipid release and exposure to pro-oxidants such as oxygen and transition metals. Under these conditions, thermal oxidation reactions predominate, in which hydroperoxide decomposition and free radical formation are accelerated, intensifying the degradation of unsaturated fatty acids [79]. In addition, protein denaturation reduces the antioxidant capacity of the system, further promoting oxidative reactions. Although microwaves do not act directly as a source of photochemical radiation, the induced structural changes may increase lipid susceptibility to photo-oxidation, particularly in the presence of photosensitizers such as heme pigments, which are capable of generating highly reactive singlet oxygen. As a result, greater formation of secondary lipid oxidation products is observed, compromising the stability and quality of meat products [79,80].

Significant color changes were also observed, including increased L* and b* values and decreased a*, indicating myoglobin denaturation and changes in the pigment redox state. These results corroborate the impact of rapid microwave heating on the stability of meat pigments [51,76]. Overall, the findings indicate that microwave processing should not be simplistically classified as either beneficial or detrimental in terms of technological properties. The literature points to a balance between operational advantages, such as reduced processing time, and potential drawbacks related to water retention, texture, color, and oxidative stability. Thus, the evaluation of microwave technology in meat systems must simultaneously consider product type, sample geometry, fat content, and the presence of free water, as these factors ultimately determine the extent of the observed physicochemical changes [75].

### 6.3. Effect of Microwave Heating on Microbiological Quality

Microwave technology can make a significant contribution to microbiological safety, primarily through the inactivation of fungi and bacteria in shorter times than those typically required for conventional heating [48].

The sterilization and preservation effects of microwave processing are associated with the combined action of thermal and non-thermal mechanisms [81], which may promote microbial inactivation through rapid heating and structural damage to cellular components, including proteins, plasma membranes, and nucleic acids, thereby compromising cell viability [82].

Microbial sensitivity to microwave radiation varies according to cellular structure. In general, Gram-negative bacteria tend to be more susceptible due to their thinner cell walls, whereas Gram-positive bacteria may exhibit greater resistance due to their thicker peptidoglycan layer [82]. However, the microbiological efficiency of the process is strongly influenced by several factors, including applied power, exposure time, frequency, product composition, water and fat content, sample geometry, and, critically, heating uniformity [67]. From a microbiological perspective, one of the main advantages of microwave heating in meat systems is its ability to reduce pathogens of public health concern, such as *Salmonella typhimurium*, *Escherichia coli* O157:H7, *Listeria monocytogenes*, and *Staphylococcus aureus* [83]. In experimentally contaminated Turkish-style meatballs, microwave treatment reduced total aerobic bacteria by approximately 3–4 log CFU/g and inoculated pathogens by 4–7 log CFU/g. Increasing microwave power and processing time enhanced microbial inactivation but also caused drying, surface darkening, and crust formation, highlighting the need to balance microbiological safety with product quality. Nevertheless, heating the meatballs at 600 or 900 W for 3 min was reported to improve consumer acceptability while providing effective microbial inactivation [83].

Similar findings have been reported for emulsified meat products and ground meats. In ground pork loin subjected to microwave heating at 915 MHz, a holding time of 15 s after reaching 100 °C resulted in pathogen reductions exceeding 5 log under power levels of 3–5 kW [84]. In this study, increasing power was also associated with changes in transmembrane potential and extensive bacterial DNA damage, indicating that the observed lethality is not solely due to bulk heating but also to severe cellular damage in exposed microorganisms [16]. Furthermore, exposure to electromagnetic fields may also inhibit metabolic pathways, reduce the expression of virulence factors, and impair microbial adaptability, reinforcing the structural and functional impact on microbial cells [82]. Despite these promising results, evidence suggests that short or non-uniform exposures may be insufficient to ensure consistent microbiological control in meat systems.

Microwave thawing can reduce processing time, energy consumption, and, in some cases, microbial growth by shortening the time the product remains within temperature ranges favorable for bacterial proliferation [85]. However, Park and Kim [86] reported higher bacterial counts in microwave-thawed samples, suggesting that thermal heterogeneity may compromise microbiological safety when the process is not properly optimized for product size and thickness [86]. Overall, the effects of microwave processing depend on product characteristics and processing conditions, which determine the extent of changes in water retention, texture, color, and oxidative stability [87].

### 6.4. Industrial Applicability of Microwave Processing

Microwave processing has progressed beyond laboratory-scale research, with continuous and pilot-scale systems available for applications such as tempering, thawing, pasteurization, sterilization, and cooking of food products [66,87]. In meat processing, microwave technology offers potential advantages including shorter processing times, rapid volumetric heating, increased processing flexibility, and improved productivity. However, the transition from laboratory or pilot-scale applications to broader industrial implementation depends on the availability of scalable and reliable equipment, compatibility with existing processing lines, and consistent control of heating uniformity under commercial conditions.

Economic feasibility is another important consideration for industrial adoption. Although microwave processing can reduce processing time and potentially improve energy and resource efficiency, its economic performance depends on equipment configuration, processing capacity, product characteristics, and operating conditions. Therefore, techno-economic assessments under industrially relevant conditions are necessary to determine whether the potential gains in processing efficiency offset equipment and operational costs. Recent advances in microwave system design and energy management may further contribute to improving the sustainability and efficiency of industrial applications [87,88].

Consumer acceptance is an additional consideration. Although microwave heating is widely familiar to consumers through domestic applications, perceptions regarding nutritional quality and food safety may influence acceptance of microwave-processed foods. Transparent communication supported by scientific evidence regarding product safety, quality, and processing performance may therefore contribute to consumer confidence and market acceptance [89]. Overall, microwave processing has reached a relatively advanced technological stage in food processing, but its broader application in the meat industry will depend on further validation of process uniformity, economic feasibility, operational reliability, and consumer acceptance under commercial conditions.

## 7. Ohmic Heating

### 7.1. Operating Principle of Ohmic Heating Technology

Ohmic heating is a thermal processing technology based on passing an electric current through food, promoting internal heating due to the Joule effect. In this process, the food acts as an electrical resistance, allowing the direct conversion of electrical energy into heat. In meat products, the major advantage of this heating approach is volumetric heating, in which heat is generated uniformly throughout the product, unlike conventional methods that transfer heat from the surface toward the interior. This characteristic contributes to greater thermal efficiency and better preservation of the sensory and nutritional properties of food [90].

The efficiency of ohmic heating processing is directly related to the electric field intensity, temperature, as well as the composition and physicochemical properties of the food. Electrical conductivity increases linearly with food temperature and ion concentration, but is inversely proportional to fat content, since lipids act as electrical insulators [91]. Regions with higher electrical conductivity tend to allow greater current passage and, consequently, greater heat generation, resulting in non-uniform heating when there are significant differences between the system components [92].

### 7.2. Effect of Ohmic Heating Technology on Meat Products

**Figure 6:**
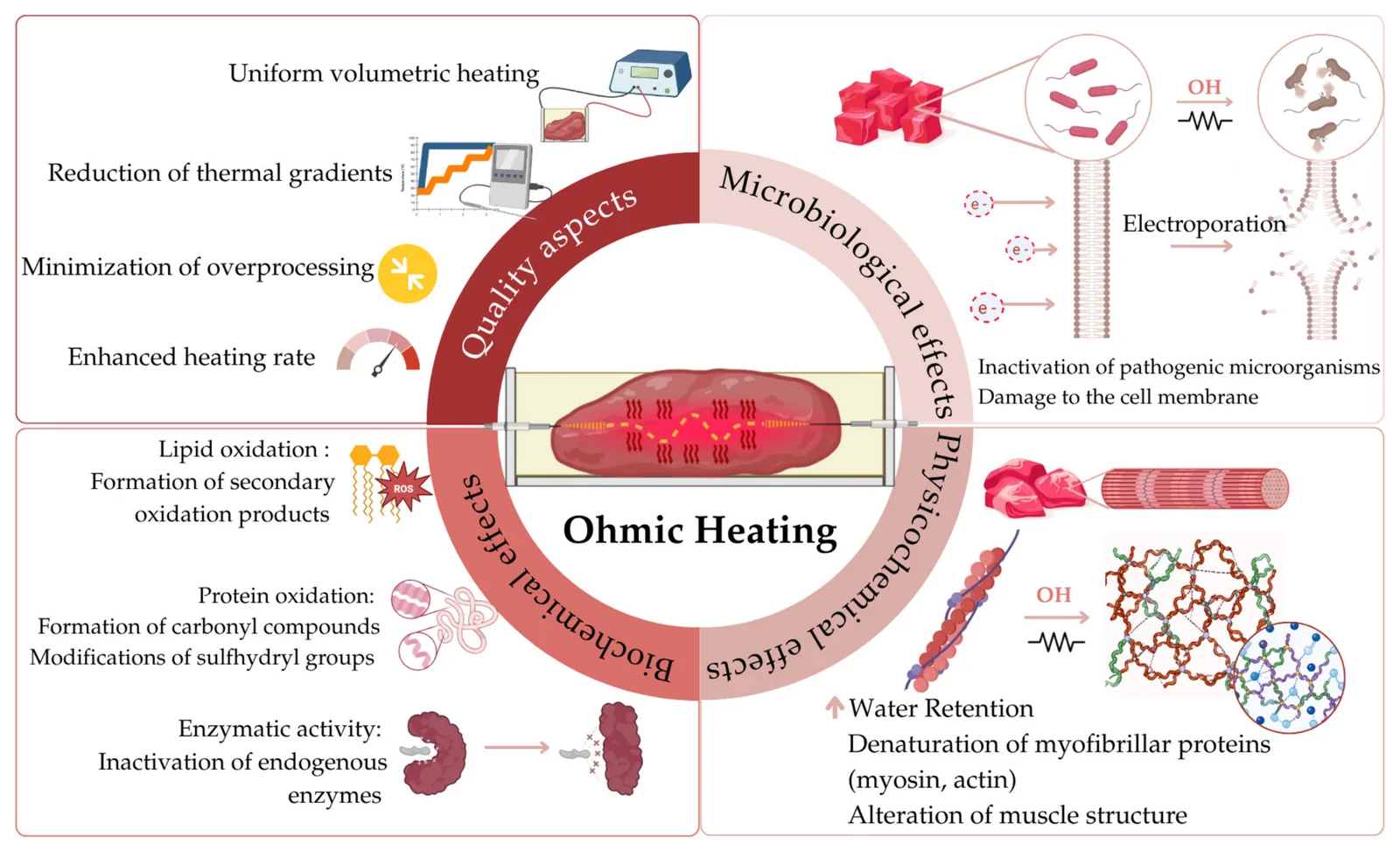
Effects of ohmic heating on meat products.

Ohmic Heating Technology can induce complex changes in the physicochemical, microbiological, sensory and technological aspects of meat products, which can be summarized in Figure 6.

The mechanisms by which ohmic heating influences the overall quality of meat products are discussed in the following sections.

#### 7.2.1. Effect of Ohmic Heating on Physicochemical and Techno-Functional Properties of Meat Products

The use of ohmic heating in meat products has been associated with direct modifications in the physicochemical and techno-functional characteristics of the resulting products. Modifications in the conformation of myofibrillar proteins may occur due to protein denaturation and reorganization, which can favor the formation of more stable three-dimensional networks with greater water retention capacity. In addition, ohmic heating promotes the exposure of hydrophobic groups and the formation of intermolecular interactions, which influence the gelling capacity of meat proteins. Chemically, this phenomenon involves the formation of carbonyl groups interacting with disulfides formed by free thiol groups. This cross-linking interaction is responsible for reducing cooking losses, favoring tenderness and juiciness [93,94].

Muscular modifications can be also observed, altering the contraction process of the fibers and forming intermolecular spaces. Unlike conventional methods, ohmic heating causes less destruction of myofibrils, due to the rate at which the food is heated, minimizing the exposure of the raw material to excessive thermal stress [95]. Therefore, the muscle structure becomes more compact and firmer due to the uniform denaturation of proteins throughout the meat, preventing the formation of fissures and cracks. Guo et al. [95] reported that heating becomes more efficient when the passage of electric current occurs parallel to the muscle fibers. This occurs due to the formation of an ordered flow through the muscle tissue, which favors the maintenance and longitudinal organization of the muscle fibers.

The main effects of ohmic heating on the structure and technical–functional properties of meat products, as well as the applied electric field conditions and temperature, are presented in Table 6.

The electrical field applied in meat processing through ohmic heating ranged from 1,67 V/cm to 70 V/cm, with maximum processing temperature reached varying from 65 °C to 105 °C (Table 6). Most studies presented indicate that ohmic heating promotes improvements in techno-functional properties, especially related to the reduction in cooking losses and greater structural uniformity of meat products. This behavior is associated with volumetric heating, which reduces thermal gradients and favors more homogeneous protein denaturation, maintaining color and hardness [91]. The application of electric fields can cause alterations in cell structure, increasing membrane permeability and favoring the release of intracellular compounds. However, the results are not entirely consistent across studies, being strongly dependent on processing conditions, such as electric field intensity and maximum temperature reached. Ohmic heating can reduce meat thawing time by up to 28–86%, decreasing drip loss from 0.01% to 1.65%, in addition to better preserving protein structure and improving water retention capacity [14]. Thus, the technology has demonstrated potential for cooking, pasteurization, thawing, extraction, and the processing of liquid and semi-solid meat products, where rapid volumetric heating may improve process efficiency while limiting excessive thermal exposure.

According to the studies evaluated, several recurring effects of ohmic heating were identified, although their magnitude varied according to the meat matrix and processing conditions. Improvements in yield, color stability, texture, and product firmness were reported in different meat systems. In addition to these technological attributes, some studies reported greater preservation of cellular integrity and improved muscle-fiber organization following processing [102]. This structural preservation has been associated with reduced mechanical stress and fissure formation during heating, potentially contributing to the maintenance of product quality [102]. Nevertheless, most studies have focused on quality immediately after processing, whereas relatively few have evaluated the stability of these characteristics during storage. This limitation highlights the need for longer-term studies to determine whether the quality benefits observed immediately after ohmic processing are maintained throughout the product’s intended shelf life.

Furthermore, several considerations should be taken into account regarding ohmic heating equipment for food, particularly meat products. The electrodes employed should be of high quality, non-corrosive, cost-effective, and highly conductive. The selection of electrode material is of critical importance, with stainless steel being the most commonly used due to its food-grade status and low susceptibility to corrosion when in direct contact with food. An increase in electrode thickness results in a lower rate of temperature rise because the greater electrode mass is associated with lower electrical resistance of the material [90,92]. Although ohmic heating is recognized for its rapid and uniform volumetric heating, the electrical energy required to cook raw meat products from the uncooked state can be considerable, representing an important limitation for process economics and industrial implementation [93].

#### 7.2.2. Effect of Ohmic Heating on Microbial Inactivation

The effectiveness of ohmic heating in microbial inactivation is related, among other mechanisms, to the phenomenon of electroporation (or electropermeabilization) of cell membranes, which can lead to the rupture and lysis of microorganisms present in the meat matrix [103]. The phenomenon of electroporation occurs due to the application of an external electric field, which induces a transmembrane potential in cells. This occurs because microorganisms naturally possess positively charged ions, such as K^+^ and Na^+^, as well as proteins with a net negative charge; when an electric potential is applied, there is movement of these charged species. When this potential reaches a critical value (~1V), the reorganization of membrane lipids occurs, resulting in the formation of hydrophilic pores. These pores increase cell permeability, allowing the flow of ions and molecules, which can lead to loss of homeostasis, leakage of intracellular contents, and, in irreversible cases, cell lysis. In addition, the application of the electric field can affect biochemical reactions by altering the molecular arrangement, modulating enzymatic activity [104].

The combination of the thermal effect with the electric field can intensify the inactivation of microorganisms, including highly resistant bacterial spores. This process occurs due to the increased mobility of ionic compounds with rising temperature, especially the calcium–dipicolinic acid (Ca-DPA) complex present in the spore nucleus, responsible for thermal resistance and stability of the vegetative form. This complex, accounting for approximately 5 to 15% of the spore dry weight, is associated with nucleus dehydration and resistance, and its release is indicative of structural damage. It can induce spore germination, making them more susceptible to subsequent thermal inactivation [105].

Studies indicate that ohmic heating can promote an additional inactivation of up to 2.4 logarithmic cycles compared to conventional heating, suggesting structural changes in cellular components and the release of intracellular compounds [105].

In addition to the way microorganisms resist in the medium, it is known that each species reacts uniquely to the applied heat treatment, requiring different combinations for its inactivation. Therefore, increasing the current intensity becomes more effective in molds, yeasts, and mesophilic bacteria. Furthermore, the matrix in which these microorganisms are present is also relevant, since foods with a high fat content can be challenging, as they heat up more slowly due to low conductivity, leading to non-uniformity in processing and the emergence of cold spots where pathogens can survive treatment [102].

Additionally, strategies to improve heating uniformity have been proposed, such as controlling electrode temperature and adjusting the electrical properties of the system. Astrain-Redín et al. [106] demonstrated that preheating the electrodes can significantly reduce thermal gradients and improve the uniformity of microbial inactivation, reducing the time required to reach safe processing levels. These approaches are essential for supporting the industrial application of ohmic heating in meat products while ensuring product quality and safety.

Regarding microbial inactivation, the literature still presents important methodological discrepancies, particularly concerning the experimental configuration used to evaluate microbial kinetics. One important issue is the size of the ohmic cell. Some studies suggest that large-volume ohmic cells may involve prolonged come-up times, potentially overestimating microbial inactivation because microorganisms are exposed to elevated temperatures before the target treatment conditions are reached. Accordingly, the use of micro-ohmic cells has been proposed to improve temperature control and provide kinetic data that more accurately reflect microbial inactivation under defined treatment conditions. Conversely, larger-volume cells may provide a more realistic representation of processing conditions at larger scales and may therefore be useful for evaluating process performance under conditions closer to industrial operation. This methodological divergence is particularly relevant for technology transfer, because laboratory-scale kinetic data may not directly predict microbial inactivation under industrial processing conditions. Therefore, the lack of consensus regarding the most appropriate experimental configuration highlights the need for standardized protocols that allow meaningful comparison among studies and more reliable extrapolation to larger-scale processing [107].

#### 7.2.3. Industrial Applicability of Ohmic Heating

Recent research on ohmic heating has progressively expanded from fundamental studies of heating mechanisms toward process optimization, engineering design, mathematical modeling, energy efficiency, and scale-up. Studies such as Tian et al. [93] and Llave et al. [96] focused on understanding the mechanisms governing ohmic heating, including protein structural modifications, electrical conductivity, and heat transfer characteristics in beef muscles. These studies demonstrated the potential of volumetric heating to improve temperature uniformity while maintaining meat quality compared with conventional thermal treatments. Similarly, Tian et al. [93] and Inmanee et al. [98] investigated microbial inactivation and post-pasteurization, supporting the potential of ohmic heating as a preservation technology. Subsequent studies have expanded the scope of evaluation to quality-related aspects, including lipid oxidation and the formation of polycyclic aromatic hydrocarbons, indicating that ohmic heating may reduce some undesirable chemical changes associated with conventional cooking [97].

More recently, research has increasingly addressed engineering and process-design aspects. Javed et al. [92], for example, highlighted system design, numerical modeling, energy analysis, and scale-up strategies, indicating a shift toward addressing the engineering requirements for industrial application. Studies outside the meat sector, including those by Icier et al. [91] and Guo et al. [95], have also contributed to the understanding of electrical conductivity behavior and moderate electric field effects in food systems, providing knowledge that may support the development of ohmic processes for complex food matrices.

Despite these advances, the industrial maturity of ohmic heating in meat processing remains limited compared with more established non-conventional technologies such as HHP. Most studies are still conducted at laboratory or pilot scale under controlled conditions, often using relatively homogeneous products. Important challenges include the heterogeneous electrical conductivity of meat matrices, electrode durability and material selection, process standardization, continuous-processing design, energy requirements, techno-economic feasibility, and regulatory validation. Further research should therefore prioritize industrial-scale demonstrations, long-term operational assessment, energy and cost optimization, and life-cycle analysis to determine the feasibility of integrating ohmic heating into commercial meat-processing operations.

## 8. Limitations of Non-Conventional Technologies

The large-scale application of non-conventional technologies still faces significant challenges. Most studies available in the literature have been conducted at laboratory or pilot scale, using relatively small volumes and highly controlled experimental conditions. The transfer of these results to industrial systems presents significant technical challenges. The initial investment in equipment, as well as operating and maintenance costs, can be high, particularly when the expected productivity gains are insufficient to offset the additional investment. Furthermore, the effectiveness of non-conventional technologies depends on the control of multiple interrelated operational parameters and the characteristics of the food matrix. The need to optimize these parameters, along with the lack of widely established standardized guidelines, represents additional challenges for defining ideal and reproducible conditions across different industrial applications [108]. Furthermore, consumer perception remains a significant barrier to the adoption of non-conventional technologies. Despite scientific validation, unfamiliar terminology and limited information can generate concerns about artificiality or toxicity [45].

Although non-conventional technologies are widely associated with improvements in the physicochemical and sensory properties of foods, undesirable effects may arise when they are applied under inappropriate processing conditions.

The use of cold plasma in meat and meat products still presents a set of technical and qualitative limitations. The abundance of reactive oxygen and nitrogen species can accelerate the oxidation of fats and proteins, which may generate undesirable volatile compounds, as well as rancid flavor and aroma [14,109,110]. In addition, cold plasma is essentially a surface treatment. Reactive species have limited penetration into the meat matrix, which means that microorganisms located below the surface or within processed products may not be inactivated. Irregularities, fissures, and the presence of skin in meats can act as physical barriers, protecting bacteria from direct contact with the reactive species generated during processing. At the same time, although it is a non-thermal technology, prolonged operation of systems such as DBD can increase the temperature of the electrodes, which in turn raises the temperature of the sample and may be sufficient to cause undesirable thermal discoloration [13,16,18,22].

HHP application in meat products processing presents important limitations, including high capital cost, batch processing constraints, and potential impacts on color and other quality attributes. Color changes are particularly evident at higher pressures (≥400–600 MPa), which may compromise consumer acceptance of fresh products [28]. At moderate pressures (150–300 MPa), color alterations are less pronounced, but protein denaturation and textural changes may still occur [26,27]. Therefore, successful industrial implementation depends on balancing processing intensity, product characteristics, and market demands, while broader commercial adoption requires coordinated efforts among researchers, meat-processing companies, equipment manufacturers, and regulatory authorities to optimize processes at industrial scale, evaluate techno-economic feasibility, and address regulatory requirements.

In ultrasound treatment, acoustic cavitation can promote the formation of reactive species and accelerate lipid oxidation reactions, thereby impairing oxidative stability and contributing to the generation of undesirable volatile compounds. In addition, sensory changes, including metallic and burnt off-flavors, have been attributed to component degradation and protein denaturation. Changes in color and texture may also occur, particularly under more severe processing conditions, highlighting the need for careful optimization of operational parameters [49]. The heterogeneity in the propagation of acoustic waves during ultrasound treatment may lead to localized variations in cavitation intensity, thereby compromising process reproducibility and efficiency at the industrial scale. In this context, the development of more efficient industrial ultrasonic reactors with improved control over energy distribution remains an active area of research and technological advancement [64].

One of the main obstacles to the use of microwave processing is the formation of non-uniform heating regions, characterized by the occurrence of hot and cold spots, resulting from the heterogeneous distribution of the electromagnetic field, product geometry, and local changes in dielectric properties throughout the process. Under more critical conditions, this behavior may evolve into thermal runaway, in which initially hotter regions absorb even more energy, leading to a disproportionate local acceleration of heating [92]. Consequently, heating non-uniformity can result in under- or over-processed regions, compromising both microbiological safety and the quality of the final product [93].

Regarding ohmic heating technology, high-intensity and/or non-uniform electric fields can also generate overheating points, resulting in regions with inconsistent texture and flavor. In addition, food contamination caused by electrode corrosion or metal solubilization under certain pH conditions can be a concern, highlighting the need to develop effective strategies to prevent fouling and metal migration. To mitigate these problems, alternating current can be used in combination with alternative electrode materials, particularly titanium, which has shown favorable performance. In addition, the application of high-frequency currents (12–300 kHz) can further inhibit Faradaic reactions, thereby improving process efficiency and electrode stability [102].

Furthermore, it should also be noted that foods with high fat or oil contents may be difficult to process by ohmic heating because of their low electrical conductivity. In addition, pathogenic bacteria present inside fat globules may be less exposed to heat treatment than those located outside these particles. A thorough understanding of the effects of the electric field on mass transfer properties, the determination of cold spots, and the overall process design is a prerequisite for establishing effective industrial processes [49].

From an implementation perspective, the transition of non-conventional technologies from laboratory research to industrial processing should be guided by a technology-specific optimization framework rather than by the maximization of a single processing effect. Process conditions should be optimized according to measurable performance indicators, including microbial reduction, shelf-life extension, product quality, processing time, energy and water consumption, yield, and the use of additives. These performance indicators should then be evaluated together with equipment capacity, capital and operating costs, maintenance requirements, process throughput, and compatibility with existing production lines. Such an approach allows the identification of processing conditions in which the technological benefit is sufficient to offset the additional costs associated with implementing the technology. In practice, the most relevant industrial applications are therefore not necessarily those producing the greatest technological effect, but those in which an optimized treatment provides a measurable advantage over conventional processing at an economically viable operating scale.

In summary, the industrial implementation of non-conventional technologies will depend on the identification of technology-specific applications in which their performance advantages justify the associated investment and operational requirements. Advances in equipment design, process modeling, continuous processing, energy efficiency, harmonized regulatory frameworks, and integration with complementary technologies will be essential to reduce implementation risks and facilitate technology transfer. Strengthening collaboration among researchers, meat-processing companies, equipment manufacturers, and regulatory authorities will also be critical for validating optimized processes under commercial conditions and establishing economically viable routes for adoption.

## 9. Future Perspectives

The future development of non-conventional processing technologies for meat and meat products should increasingly move from proof-of-concept studies toward process validation, scale-up, and integration into industrial processing systems. The integration of these technologies with advanced digital control systems, real-time monitoring, computational modeling, and artificial intelligence may improve process optimization, reproducibility, and control of critical processing parameters. In this context, future research should prioritize experimental approaches that generate data under industrially relevant conditions and enable reliable comparison among different processing strategies.

For cold plasma, future research should focus on optimizing treatment conditions to maximize microbial safety while minimizing undesirable effects on color, lipid oxidation, and sensory quality. Particular attention should be given to the development of scalable treatment systems and the integration of cold plasma with complementary technologies, including plasma-activated water, ultrasound, edible films, and natural antimicrobials. Such approaches may facilitate the incorporation of plasma into existing meat-processing operations while supporting clean-label preservation strategies.

For HHP, future research should move beyond empirical optimization toward mechanistically informed and system-oriented approaches. A key priority is the quantitative understanding of protein–water interactions under pressure, particularly the transition between unfolding and aggregation regimes. Systematic studies linking pressure, time, temperature, product composition, and functional outcomes are also needed to establish transferable processing windows across different meat matrices. Further validation of HHP combined with natural antimicrobials and other hurdle strategies, together with consumer acceptance studies, may support the expansion of its applications while maintaining product quality.

For ultrasound, future research should prioritize the development of efficient continuous-flow reactors capable of generating more homogeneous acoustic fields in complex meat matrices. Greater understanding of cavitation behavior, process scale-up, energy efficiency, and real-time monitoring will be essential for improving reproducibility and industrial performance. Particular attention should also be given to applications in which ultrasound provides a clear advantage in process intensification, such as marination, thawing, tenderization, drying, and mass transfer, rather than relying solely on its isolated antimicrobial effect.

For microwave processing, future research should focus on improving heating uniformity in heterogeneous meat matrices through advances in electromagnetic modeling, real-time temperature monitoring, and intelligent process control. Pilot- and industrial-scale studies should evaluate energy efficiency, product quality, process reliability, and compatibility with existing processing lines to determine the conditions under which microwave processing provides a clear advantage over conventional heating.

For ohmic heating, future research should prioritize the development of standardized experimental protocols, improved understanding of electrical conductivity in heterogeneous meat matrices, optimization of electrode materials and configurations, and validation of continuous and large-scale processing systems. Particular attention should be given to energy requirements, electrode durability, microbial inactivation kinetics, and techno-economic feasibility. Studies conducted under industrially relevant conditions will be essential to determine the specific meat-processing applications in which ohmic heating can provide a meaningful advantage over established thermal processes.

Across all technologies, future research should increasingly incorporate techno-economic analysis, life-cycle assessment, regulatory considerations, and consumer acceptance alongside technological performance. Greater collaboration among researchers, meat-processing companies, equipment manufacturers, and regulatory authorities will be essential to facilitate technology transfer, process standardization, and industrial validation. Importantly, future studies should prioritize applications in which non-conventional technologies provide measurable advantages in product quality, safety, processing efficiency, sustainability, or clean-label formulation, thereby increasing the likelihood that promising laboratory findings can be translated into economically viable and commercially relevant meat-processing solutions.

## 10. Final Considerations

Non-conventional processing technologies, including cold plasma, high hydrostatic pressure, ultrasound, microwave processing, and ohmic heating, have demonstrated significant potential to improve the quality, safety, functionality, and sustainability of meat and meat products. Despite operating through distinct mechanisms, these technologies share the potential to enhance processing efficiency, reduce reliance on conventional thermal treatments, and support the development of clean-label products.

The literature indicates that cold plasma, high hydrostatic pressure, ultrasound, and ohmic heating can improve microbial safety and techno-functional properties, whereas microwave processing offers important advantages related to rapid volumetric heating and reduced processing times. However, the application of these technologies may also result in undesirable effects, including lipid oxidation, color deterioration, texture modifications, and heating non-uniformity, depending on processing conditions and product characteristics. Therefore, technological benefits must be evaluated in conjunction with potential impacts on product quality, processing costs, and consumer acceptance.

For these technologies to be effectively implemented at industrial scale, further studies are required to evaluate their economic feasibility through cost–benefit analyses, return on investment (ROI), payback period, energy and water consumption, maintenance costs, and production-line productivity. In addition, the development of robust industrial equipment capable of continuous operation, high processing capacity, clean-in-place (CIP) compatibility, low maintenance requirements, and seamless integration into automated processing lines is essential. Process parameters, including power, frequency, processing time, electric field intensity, and target temperature, should also be standardized to improve process reproducibility and facilitate technology transfer.

Another important strategy is the integration of non-conventional technologies with established processing methods rather than their complete replacement. Such hybrid approaches may reduce implementation costs and technological risks while maximizing the advantages of complementary preservation mechanisms. Strengthening collaborations between universities, food-processing companies, equipment manufacturers, and regulatory authorities will also be essential for industrial validation, prototype development, process standardization, and adaptation of these technologies to the specific requirements of different meat-processing facilities. Harmonized regulatory frameworks and robust consumer acceptance data will further support technology transfer and commercial adoption.

Taken together, the evidence indicates that the technologies reviewed are not at the same stage of technological maturity. HHP currently presents the strongest evidence of commercial implementation in meat products, particularly for post-packaging treatment of ready-to-eat products, whereas ultrasound and microwave processing have reached industrial or pilot-scale applications for selected operations. Cold plasma and ohmic heating remain less mature in meat processing, despite substantial advances in process optimization and engineering development. Therefore, future research should not focus solely on demonstrating additional technological effects, but rather on determining where each technology provides a clear advantage over established processing methods and whether such advantages can be translated into economically viable, standardized, and regulatory-compliant industrial processes.

From a technology-specific perspective, further industrial development should prioritize applications that match the strengths of each technology with clearly defined processing needs. For HHP, opportunities are particularly associated with post-packaging treatment of ready-to-eat meat products and the extension of shelf life while preserving product quality. For ultrasound, priority should be given to process-intensification applications such as marination, tenderization, thawing, and mass-transfer enhancement, where its benefits can complement rather than replace conventional operations. Microwave processing should focus on applications in which rapid heating or thawing provides a measurable productivity advantage, together with improved control of heating uniformity. For cold plasma, surface decontamination, plasma-activated fluids, and integration with hurdle technologies represent promising routes for incorporation into existing processing steps. For ohmic heating, applications involving high-throughput cooking, pasteurization, or liquid and semi-solid meat systems may provide more favorable conditions for exploiting rapid volumetric heating while overcoming some of the limitations associated with heterogeneous whole-muscle matrices.

In this context, further research should be prioritized toward applications in which the technology provides a measurable advantage in product quality, safety, processing efficiency, sustainability, or clean-label formulation, rather than toward further proof-of-concept studies with limited potential for scale-up. Ultimately, future progress will depend on shifting research priorities from demonstrating technological effects under laboratory conditions toward validating robust, economically viable, scalable, and industry-relevant processing solutions under pilot and commercial conditions. This transition will require integrated evaluation of technological performance, economic feasibility, regulatory compliance, resource efficiency, and consumer acceptance. Overall, non-conventional processing technologies represent valuable tools for the modernization of the meat industry, but their long-term impact will depend on the ability to translate technology-specific advantages into practical and economically justified industrial applications.

## Figures and Tables

**Table 1:** Effects of cold plasma on the physicochemical and techno-functional properties of meat and meat products.

Meat Product	Type of Plasma/Gas Used	Process Parameters	Results	References
Meat product	Plasma-activated water (PAW) via atmospheric plasma jet (APPJ)/Atmospheric air	Time: 1 min; Voltage: 600 W	L* (surface): ↑; POV: ↑; Sulfhydryls: ↓; WHC (thawing loss): ↑; WHC (cooking loss): ↓; Hardness: ↓	[12]
Chicken breast	DBD/Dry air	Time: 1, 3 and 5 min; Voltage: 100 kV; Frequency: 60 Hz	pH: ↓; WHC: ↓ (after 5 min)	[13]
Pork loin	DBD/O_2_, N_2_, CO_2_	Time: 60 s; Voltage: 85 kV; Distance: 40 mm	b*: ↑; MetMb: ↑; TBARS: ↑; Carbonyls: ↑; Sulfhydryl groups: ↓	[14]
Dried meat	Plasma-activated brine (PAB)/N_2_	Time: 10 min activation; Frequency: 20 kHz	pH: ↓ (air); a* (air): ↑; Residual nitrite: ↑; Texture (N_2_): ↓	[15]
Chicken meat (breast and thigh)	Plasma-activated acetic acid (PAAA) via DBD/Atmospheric air	Time: 30 min activation; Voltage: 8.4 kVpp; Frequency: 2.2 kHz	pH: ↓; L* (breast): ↑; a* (breast): ↓; b* (breast): ↓; TBARS: ↓	[16]

Note: DBD: Dielectric Barrier Discharge; PAW: Plasma-activated water; PAB: Plasma-activated brine; PAAA: Plasma-activated acetic acid; WHC: Water-holding capacity; TBARS: Thiobarbituric acid reactive substances; POV: Peroxide value; ↑: Significant increase (*p* < 0.05); ↓: Significant decrease (*p* < 0.05).

**Table 2:** Effect of cold plasma on the microbial decontamination of meat products.

Meat Product	Type of Plasma/Gas Used	Process Parameters	Microbial Load Reduction (log)	References
Beef burgers	In-package DBD combined with protein film and essential oil	Time: 10 min; Voltage: 70 kV	0.91 log CFU/g (from 4.66 to 3.75)	[20]
Chicken breast fillets	Direct DBD/Argon gas	Time: 3–5 min; Voltage: 20 kV	> 2.0 log10 CFU/g (APC)	[18]
Beef (frozen)	Thawing with plasma-activated water	Time: 1 min; Voltage: 600 W	TVC: 1.62 log; Fungi and yeasts: 1.76 log	[21]
Fresh chicken breast	In-package atmospheric cold plasma	Time: 5 min; Voltage: 100 kV	~1.5 to 2.0 log (mesophiles, psychrotrophs, and *Enterobacteriaceae*)	[12]
Fresh pork loin	In-package dielectric barrier discharge (DBD	Time: 60 s; Voltage: 85 kV	Reductions up to 0.38 log initially; effectiveness increases with O_2_	[14]
Cured beef	Plasma-activated brine	Time: 10 min; Voltage: 20 kV	0.5 log in brine; 0.85 log in final product (*L. innocua*)	[15]
Chicken breast and thigh	Plasma-activated acetic acid	Time: 30 min; Voltage: 8.4 kVpp; Frequency: 2.2 kHz	0.98 log (breast) and 1.19 log (thigh) additional vs. pure acetic acid (*S. Typhimurium*)	[16]

DBD: Dielectric Barrier Discharge; CFU: Colony-Forming Units.

**Table 3:** Comparative analysis of HHP processing conditions and effects on meat systems.

Meat Matrix	Pressure/Conditions	Target Outcome	Observed Effects	Mechanism	Response Pattern	Limitation	Study
Deer	150–600 MPa for 5 min	Texture	↑ firmness	Structural disruption	Disruption-dominant	Color	[28]
Sausage	200 MPa for 10 min	Clean-label	↑ firmness, ↓ cook loss	Protein unfolding	Functional-dominant	Saltiness	[30]
Sausage	150 MPa for 5 min	Clean-label	↑ WHC	Network formation	Functional-dominant	Texture	[31]
Fermented Dry-cured sausage	600 MPa for 8 min	Shelf-life	↑ lipid and protein oxidation; changes in color	Structural exposure	Oxidative-dominant	Oxidation	[32]

↓: Statistically significant reduction (*p* < 0.05); ↑: Statistically significant increase (*p* < 0.05).

**Table 4:** Applications of ultrasound in meat processing: mechanisms, technological effects, and industrial benefits.

Application	Associated Mechanism	Main Technological Effects	Industrial Benefits	References
Meat tenderization	Myofibrillar fragmentation, sarcomere disorganization, and structural modification of muscle proteins	Reduced shear force and increased tenderness	Improved texture and meat quality	[48]
Accelerated aging	Increased cell permeability, microchannel formation, and enhanced mass transfer	Intensified post-mortem proteolysis	Reduced aging time and storage costs	[49]
Ultrasound-assisted marination	Enhanced mass transfer, microchannel formation, and increased muscle matrix permeability	Improved brine diffusion and water retention	Reduced marination time and increased yield	[50]
Emulsified products	Conformational changes in myofibrillar proteins with exposure of hydrophobic groups and increased surface hydrophobicity	Enhanced emulsifying activity and stability, with more homogeneous emulsions	Improved texture and stability of processed products	[51]
Ultrasound-assisted freezing	Enhanced nucleation and controlled ice crystal growth	Formation of smaller and more uniform ice crystals	Reduced structural damage and improved water retention	[52]
Ultrasound-assisted thawing	Enhanced heat transfer via acoustic cavitation and microstreaming	Reduced thawing time	Reduced drip loss and improved meat quality	[53]
Ultrasound-assisted drying	Intensified heat and mass transfer	Increased water removal rate	Reduced drying time and improved energy efficiency	[54]
Salt diffusion in cured meats	Enhanced mass transfer within the muscle matrix	More homogeneous NaCl distribution	Reduced curing time	[55]
Ultrasound-assisted protein extraction	Acoustic cavitation promoting protein disaggregation and increased solubility	Higher extraction yield and improved techno-functional properties	Expanded use of proteins in food systems	[56]
Extraction of bioactive compounds from meat by-products	Acoustic cavitation inducing protein matrix disruption	Increased yield and bioactivity of peptides	Valorization of industrial by-products	[57]
Fat reduction in meat products	Formation of Pickering emulsions stabilized by ultrasound-modified proteins	Improved stability and texture in low-fat systems	Development of healthier products	[58]
Enzyme inactivation	Cavitation- and heat-induced enzyme denaturation (thermosonication)	Reduced enzymatic activity	Enhanced storage stability	[59]

**Table 5:** Influence of processing conditions on the physicochemical and technofunctional properties of microwave-treated meat products.

Product	Sample Form	Processing Parameters	Main Results	Reference
Cooking (yak meat)	Cubic sample (3 × 3 × 1.5 cm)	420–700 W; 90–310 s	↑ Weight loss (37–45%); ↑ shear force; ↓ a*; ↑ volatile compounds; muscle fiber shrinkage	[70]
Cooking (beef)	Muscle steak	Internal temperature of 71 °C for 20, 30, 40, 50, or 60 s	Longer microwave cooking times increased mass loss	[71]
Cooking (beef)	Cubic sample (2 × 2 × 2 cm)	700 W for 5 min	↑ nucleotide and free amino acid content; ↑ sensory acceptability and color	[72]
Reheating (pork)	Meatballs	500 W for 2 min	↑ Lipid oxidation; ↓ water retention; ↑ hardness; steam reheating better preserved quality	[73]
Microwave-assisted brining (cured beef)	Cubic sample (10 × 10 × 2 cm)	90 °C; brining time: 105 min; microwave time: 85 s	↑ Salt diffusion; ↑ protein modifications; partial texture improvement; risk of oxidation	[74]

↓: Statistically significant reduction (*p* < 0.05); ↑: Statistically significant increase (*p* < 0.05).

**Table 6:** Main effects of ohmic heating on the structure and techno-functional properties of meat products, including the applied electric field strength and processing temperature.

Product	Sample Form	Electric Field (V/cm)	Maximum Temperature (°C)	Observed Structure Modification	Techno-Functional Properties	Results Obtained When Compared to the Conventional Heating	Reference
Cow meat (M. longissimus dorsi)	Whole muscle	12	72	Reduced protein denaturation (proteomics); reduced myofibrillar degradation	↓ cooking loss; ↓ shear force (↑ tenderness); ↑ redness (a*)	Better tenderness, less loss, and less protein damage vs. water bath.	[94]
Australian Beef	Cubic samples	50	65	The endogenous fat apparently began to melt -after OH treatment	Low cooking and shrinkage losses, good temperature uniformity, reduced time required to heat the beef muscles when increased frequencies were applied	Modifications of vertical cross-section at the center structure of beef samples were observed after OH	[96]
Beef (muscle)	Whole muscle	40–70	70–80	Voltage-dependent lipid oxidation; moderate structural changes.	TBARS ↑ with higher voltage; quality dependent on the process.	It can increase oxidation compared to isolated OH; even faster than conventional OH.	[97]
Sausage (vacuum-packed)	Emulsion product	23	75	Structure practically preserved; no significant alterations.	Stable WHC; stable pH; low lipid oxidation.	>5 log reduction in Listeria vs. 3–4 log (conventional); shorter time	[98]
Pork	Whole muscle	21	70	Microstructure: ↑ Myofibril alignment; cell preservation	WHC: ↑ Retention; Texture: ↑ Sensory firmness	Superior firmness while maintaining internal juiciness compared to pan-fry.	[99]
Conventional beef and Wagyu	Whole muscle	~1,67 V/cm	65	Limited denaturation (especially below 60 °C); formation of molten fat channels; slight alteration in connective tissue.	High quality retention; less protein denaturation; greater energy efficiency; rapid volumetric heating.	Less nutrient loss and better sensory quality; fewer “cold spots”; faster heating than conventional methods.	[96]
Chicken Sausage	Emulsion	13.5	75	Not detailed	Performance: High thermal efficiency	Faster and more even heating than a double boiler.	[100]
Bologna-style sausage	Product	3 V/cm	105	Protein network with larger fat globules (microstructure)	↑ water retention; ↓ hardness, elasticity and chewiness	Time is approximately 95% shorter; similar microbiology.	[101]

↓: Statistically significant reduction (*p* < 0.05); ↑: Statistically significant increase (*p* < 0.05).

## Data Availability

No new data were created or analyzed in this study. Data sharing is not applicable.

## Abbreviations

The following abbreviations are used in this manuscript:

Ca-DPA	calcium-dipicolinic acid
CFU	Colony-Forming Units
DBD	Dielectric Barrier Discharge
EAI	emulsifying activity index
ESI	emulsion stability index
HHP	High hydrostatic pressure
MDA	malondialdehyde
·OH	hydroxyl radicals
PAAA	Plasma-activated acetic acid
PAB	Plasma-activated brine
PAW	Plasma-activated water
POV	Peroxide value
RNS	reactive nitrogen species
ROS	reactive oxygen species
TBARS	Thiobarbituric acid reactive substances
US	Ultrasound
WHC	Water-holding capacity
